# Supplementary material for: Dual Costimulatory and Coinhibitory Targeting with a Hybrid Fusion Protein as an Immunomodulatory Therapy in Lupus Nephritis Mice Models
Source: Int J Mol Sci. 2022 Jul 29;23(15):8411. doi: 10.3390/ijms23158411 (PMC9369380; doi:10.3390/ijms23158411)
Supplement: Supplementary file 1 [file ijms-23-08411-s001.zip › ijms-1812856-supplementary.pdf]

Gene	NZBWF1 model CYP	NZBWF1 model Hybri	MRL/lpr model CYP	MRL/lpr model Hybri
C1qc	↓	↓	↓↓	↓↓
C3		↓	↓↓	↓↓
Ccl7	↓	↓	↓	↓
CCR5				↓
CD14	↓	↓↓	↓	↓↓
CD19	↓			
CD28	↓			
CD3e	↓		↓	↓
CD4	↓		↓↓	↓↓
CD40	↓	↓		↓↓
CD40L	↓			
CD80				↓
CD86	↓	↓		↓
CD8A	↓	↓↓		↓↓
CTLA4				
CXCL10	↓	↓	↓↓	↓
FasL	↓	↓		↓
Fcgr2b		↓		
Fcgr3	↓	↓	↓↓	↓↓
Fcgr4			↓↓	↓
Fcnb				
FoxP3	↓		↓	↓
HGF				
IL2	↓	↓		
IL2ra				
IL6		↓		
INFg	↓			
IRF5		↓	↓	↓↓
Pdcd1			↓	↓
Pdcd11g2			↓	↓
Ptpn6	↓↓	↓↓	↓	↓↓
Ptprc	↓↓	↓		↓
RorC				
S100A6	↓	↓	↓↓	
SOX9		↓	↓	↓↓
TLR2	↓	↓↓	↓↓	↓↓
TLR4	↓	↓		↓
TLR7	↓	↓		
TNFb	↓↓	↓↓	↓↓	↓↓
Tnfrsf9	↓	↓		
Tnfsf9	↓	↓		↓↓
Tnfsfl3b	↓	↓	↓	↓↓
VcamL	↓	↓	↓↓	↓↓

Supplementary Table S1. List of all 43 genes included in the study. Significant differences for each treatment compared to Vehicle group at the end of the studies, at 36 weeks of age for the NZBWF1 model and at week 22 for the MRL/lpr model. ↓: downregulated genes $p < 0.05$; ↓↓: downregulated genes $p < 0.01$.

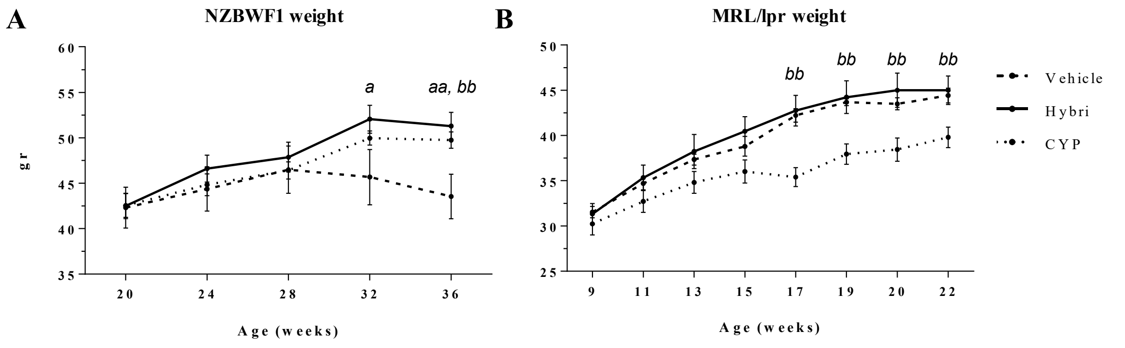

Supplemental Figure S1. (**A**) Weight values expressed in grams for NZBWF1 mice model thorough the study. Vehicle group: n = 8; Hybri group: n = 9; CYP group: n = 8. (**B**) Weight values expressed in grams for MRL/lpr mice model thorough the study. Vehicle group: n = 8; Hybri group: n = 6; CYP group: n = 6. Data are expressed as mean values ± S.E.M. Hybri Vs. Vehicle: *a* = $p < 0.05$, *aa* = $p < 0.01$; CYP Vs. Vehicle: *b* = $p < 0.05$, *bb* = $p < 0.01$.
